# Improving integrated care for (future) parents facing vulnerable circumstances in the early life course of their (future) child: An action research protocol

**DOI:** 10.1371/journal.pone.0305557

**Published:** 2024-10-31

**Authors:** S. C. Munshi, A. M. Weggelaar-Jansen, A. van den Berg-Bakker, L. M. G. Blanchette, H. W. Harmsen van der Vliet-Torij, M. W. Hodes, M. van ‘t Hof, M. P. Lambregtse-van den Berg, L. van der Meer, H. E. Ernst-Smelt, H. H. Bijma

**Affiliations:** 1 Division of Obstetrics and Foetal Medicine, Department Obstetrics and Gynaecology, Erasmus MC–Sophia Children’s Hospital, University Medical Centre, Rotterdam, South-Holland, the Netherlands; 2 Tranzo, Tilburg University, Tilburg, North-Brabant, The Netherlands; 3 Centrum voor Jeugd en Gezin Rotterdam-Rijnmond (Preventive Youth Health Care), Rotterdam, South-Holland, the Netherlands; 4 Department of Social Development, City of Rotterdam, Rotterdam, South-Holland, The Netherlands; 5 Research Centre Innovations in Care, Rotterdam University of Applied Sciences, Rotterdam, South-Holland, The Netherlands; 6 Department of Family Support Services, ASVZ, Care Organisation for People with Intellectual Disabilities, Sliedrecht, South-Holland, the Netherlands; 7 Department of Public Health and Care, Municipal Public Health Service (GGD) Rotterdam-Rijnmond, Rotterdam, South-Holland, the Netherlands; 8 Departments of Psychiatry and Child & Department of Adolescent Psychiatry, Erasmus Medical University Centre, Rotterdam, South-Holland, The Netherlands; PLOS: Public Library of Science, UNITED KINGDOM

## Abstract

**Introduction:**

Suboptimal circumstances during the early life course, ranging from 100 days before conception to 1000 days following birth, significantly impact a child’s future health and well-being. To optimize these circumstances, collaboration is needed which includes professionals working in medical, social and public domains, as well as parents. This action research protocol aims to improve care for (future) parents facing suboptimal circumstances during the early life course by enhancing inter-professional, cross-domain collaboration and (future) parents-professional collaboration. By employing iterative action research cycles, we seek to foster integrated care pathways and improve continuity of care across the medical, social and public domains. The research will be conducted in Rotterdam, the Netherlands.

**Methods and analysis:**

Four action research cycles incorporating descriptive qualitative and quantitative studies, including focus groups, questionnaires and observations with (future) parents facing suboptimal circumstances, professionals and policymakers. This intervention study will not only foster improved, integrated care around identification of the need of additional support, referral and care, but also foster the necessary conditions for a self-supporting neighbourhood care learning network of (future) parents, professionals and policymakers to encourage bidirectional feedback and enable reflection beyond a single organisation. These interventions will also be evaluated.

**Dissemination:**

The results will be disseminated through peer-reviewed publications, layman summaries, regional and national knowledge platforms and presentations and factsheets relevant to all involved actors.

## Introduction

Suboptimal circumstances during the early life course, ranging from 100 days before conception to 1000 days following birth [1], significantly impact a child’s future health and well-being [2–5]. This understanding stems from the Development Origins of Health and Disease (DOHaD) paradigm, emphasizing the long-term effects of suboptimal early life circumstances, including those experienced in utero, on later health outcomes [2]. These suboptimal circumstances are referred to in literature as poor nutrition, impaired parental health and well-being, impaired parental mental health, social determinants, and exposure to toxic substances/suboptimal environment [6]. This action research protocol aims to improve care for (future) parents facing suboptimal circumstances during the early life course by enhancing inter-professional, cross-domain collaboration and (future) parents-professional collaboration. By employing iterative action research cycles, we seek to foster integrated care pathways and improve continuity of care across the medical, social and public domains.

In the early 2010s, research into perinatal outcomes in the Netherlands highlighted disparities compared to other European countries. Besides medical factors, suboptimal environmental factors were identified as contributors to these disparities, particularly in various neighbourhoods where parents facing social and economic challenges experienced heightened stress, potentially leading to unfavourable perinatal outcomes [7–11]. Consequently, recognizing the need for social domain involvement in care, several Dutch cities initiated programs to foster inter-professional, cross-domain collaboration and integrated care pathways. These programs aim to create multidisciplinary coalitions involving representatives at various organisational levels from the medical, social and public domains as well as private organisations and the municipality in order to provide integrated care for parents facing suboptimal circumstances. These multi-sectoral teams transcend traditional role boundaries of professionals, including gynaecologists, midwives, social workers, preventive youth health physicians and nurses, mental health care professionals and other care providers. The overarching goal of the programs is to provide “*care trajectories tailored to the unique circumstances of each patient*” and ‘*designing referral systems and procedural road maps*’ [11].

Alike other cities, the municipality of Rotterdam and the Erasmus University Medical Centre have implemented various programmes to enhance inter-professional, cross-domain collaboration and integrated care for (future) parents in ‘vulnerable circumstances’. These organisations utilize the overarching term ‘vulnerable circumstances’ to describe (future) parents facing suboptimal circumstances, signifying an imbalance in which risk factors outweigh protective factors [12]. Moreover, the term is used to triage and to procure appropriate care for the multi-issues associated with suboptimal circumstances. Within this research project, risk factors for vulnerable circumstances include intellectual disabilities, substance use, psychiatric illness, unintended pregnancy, teenage pregnancy, and multiple psychosocial issues. However, to date, there is limited understanding of how women living in these vulnerable circumstances interpret the term ‘vulnerability’ [13–15]. Therefore, research into the conceptualisation of vulnerability will allow for a more profound description of the term in line with the lived experiences of these women. As such, these insights can inform especially professionals and policymakers on how to better reach out and support women in engaging with care.

Additionally, though from a biological perspective the early life course is a continuum, research shows that the provision of care in this period is still fragmented [16], especially for (future) parents facing vulnerable circumstances (FPVC). To enhance integrated care and inter-professional cross-domain collaboration, it is essential to understand how organisational fragmentation influences continuity of care (from various domains) before conception and after birth, a perspective currently lacking. Such insights from FPVC and professionals will elucidate barriers and facilitators for inter-professional and (future) parent-professional collaboration, enabling improved alignment and coordination, as well as foster integrated care around identification, referral and care in the early life continuum.

Moreover, enhancing collaboration between (future) parents and professionals requires a shared understanding of the importance of suboptimal circumstances for the future health and well-being of a child, including a comprehension of what these factors entail. Currently, there is a lack of understanding regarding the awareness of (future) parents with regard to suboptimal circumstances during early life course [4]. Professionals engaged in this period play a crucial role in communicating knowledge about suboptimal circumstances to (future) parents, making their awareness equally important. These insights can guide professionals in effectively communicating the significance of these circumstances during consultations with FPVC, as well as for policymakers to inform efforts to disseminate this scientific knowledge to the public, ultimately aiming to improve future children’s health and well-being.

As insights into the (dis)continuity of care and degree of integrated care require engagement of actors such as FPVC, professionals and policymakers, we apply action research to explore how this enfolds in practice. Thereby, we aim to keep them engaged by consistently giving feedback on the insights gained, as is common in action research. One method to achieve this is to establish self-supporting neighborhood learning networks involving FPVC, professionals and policymakers. A network can be seen as a set of actors connected by certain relationships working on a common goal [17]. Thereby, a learning network aims to generate the rapid exchange of knowledge, new applications and knowledge development and learning by doing together. Additionally, one of the principles of a successful new network structure lies in creating insight into actor perspectives and through actor participation. However, currently, little is known about how to effectively engage FPVC during the early life course in a health-care based learning network. Therefore, we aim to explore the necessary conditions to establish a sustainable, self-supporting neighbourhood learning network in which FPVC, professionals and policymakers can continuously exchange their knowledge and experiences regarding inter-professional, cross-domain collaboration, (future) parents-professional collaboration and with integrated care pathways.

## Materials and methods

### Study design

This study is a cyclic action research project. We follow the definition of action research by Coughlan and Coghlan (p. 222): “[…] action research is research *in* action, is participative, is concurrent with action and is both a sequence of events and an approach to problem solving.” [18]. Action research offers the opportunity to implement novel scientific knowledge directly, provides the flexibility needed to implement across agencies and will yield more generalizable knowledge on innovation processes [19]. We chose this method as we want to disseminate our findings to participants and actors in a multi-sectoral collaboration to encourage bidirectional feedback and enable reflection beyond single organisations as research has shown this strategy stimulates productive change and improvement in a participatory environment [20].

### Project aim and objectives

The main aim is to answer this overarching question: *How can professionals across the medical*, *social and public domains*, *along with policymakers in the municipality of Rotterdam*, *collaborate with FPVC to enhance inter-professional and (future) parents-professional collaboration*, *thereby achieving more integrated care for FPVC during the early life course*? The objectives include ensuring that:

1. FPVC and professionals will have a shared understanding of vulnerable circumstances and the importance of the early life course;
2. FPVC and professionals will collaborate in openness and trust to improve care during the early life course;
3. FPVC and professionals will collaborate to foster integrated care around identification of need for additional support, referral and care during the early life course;
4. The necessary conditions will be evaluated for a self-supporting neighbourhood care network of (future) parents, professionals and policymakers that will enable them to share knowledge, experiences and collaboratively improve care for (future) parents during the early life course.

#### Actors

The multi-sectoral collaboration known as ‘Growing Together 010’ (in Dutch: ‘Samen Groeien 010’) comprises a consortium of seven organisations in the city of Rotterdam. The ‘010’ corresponds to the regional area code of Rotterdam. Collectively, the partner institutions engaged in early life course are: municipality of Rotterdam, the inner city-based Erasmus University Medical Centre, preventive youth health care (Centrum voor Jeugd en Gezin Rotterdam-Rijnmond), social care (Moeders van Rotterdam), family planning care from the Municipal Public Health Service (Nu Niet Zwanger), care and services for persons with intellectual disabilities (ASVZ), Regional Consortium Pregnancy and Childbirth Southwest Netherlands (RCSWN, Regionaal Consortium Zwangerschap en Geboorte Zuidwest Nederland) and Tilburg University, which plays a significant advisory role in action research. The consortium operates on multiple levels, including the governing body, project team and executive professionals of all relevant domains. Additionally, the consortium benefits from its own advisory parent panel, offering guidance throughout various project phases.

#### Nomenclature

Within this paper, we use the term ‘actor’ to indicate abovementioned parties. We acknowledge that authors might often use the term ‘stakeholder’ to indicate similar involved parties. However, because of recent debates about the colonial connotations with the term ‘stakeholder’, we find it appropriate not to use this term for our protocol [21–23].

#### Project setting: Rotterdam

This study will be conducted in Rotterdam, the second largest city in the Netherlands, where approximately 7500 children are born annually. Maternity care in the Netherlands is organized into various levels based on medical risks [24]. During the early life course, several professionals from the medical domain deliver standard care, including general practitioners, midwives, and postnatal caregivers who provide medical and practical assistance regularly at home for eight days after childbirth. Thereafter, preventive youth health care, which caters to all Dutch children until the age of 18 years, delivers postnatal services in the public domain.

Beyond standard care, additional care is available during the early life course in the Netherlands. In the medical domain, this may include services for mild intellectual disabilities or addiction treatment. The public domain offers care through programs like ‘Nu Niet Zwanger’(Not Pregnant Now) for (future) family planning, while the social domain provides aid for housing or debts issues for instance through neighborhood-based teams employed by the municipality. Lastly, child protection services can intervene when concerns arise about the ability of (future)parents to adequately care for their (future) child. Therefore, collaboration among the medical, social and public domains is essential for FPVC, although differences in laws and regulations can sometimes hinder effective cooperation in practice.

Within Rotterdam, the disadvantaged neighbourhood of Delfshaven with a diverse range of socio-demographic characteristics, is selected to establish a self-supporting neighborhood learning network involving FPVC, professionals and policymakers (see Table 1). Within this neighborhood, our action research cycli will take place. Delfshaven is adjacent to the district where Erasmus MC, a city-based tertiary hospital, is located. Within Delfshaven, there are five midwifery practices, multiple maternity care practices, two locations of preventive youth health care services, and five neighborhood-based teams employed by the municipality. Additionally, the area is rich in community initiatives and hosts numerous organizations offering supplementary care, such as Antes, which aids pregnant women dealing with addiction.

**Table 1:** Descriptive characteristics of pilot district neighbourhood Delfshaven.

	Delfshaven	Rotterdam
Number of inhabitants	76.786	651.269
Household consisting of two persons and one or more children (%)	17%	19%
Standardized household income (lowest, middle, highest, %)	60%, 29%, 11%	52%, 32%, 16%
Non-Western migration background (%)	54%	39%
Preterm birth^1^	5.62%	5.87%
Small for gestational age^2^	12.92%	12.75%

^1^Singeltons between 2016 and 2021 from 24 weeks of pregnancy

^2^Singeltons between 2016 and 2021 from 24 weeks of pregnancy

## Research design

**Fig 1:**
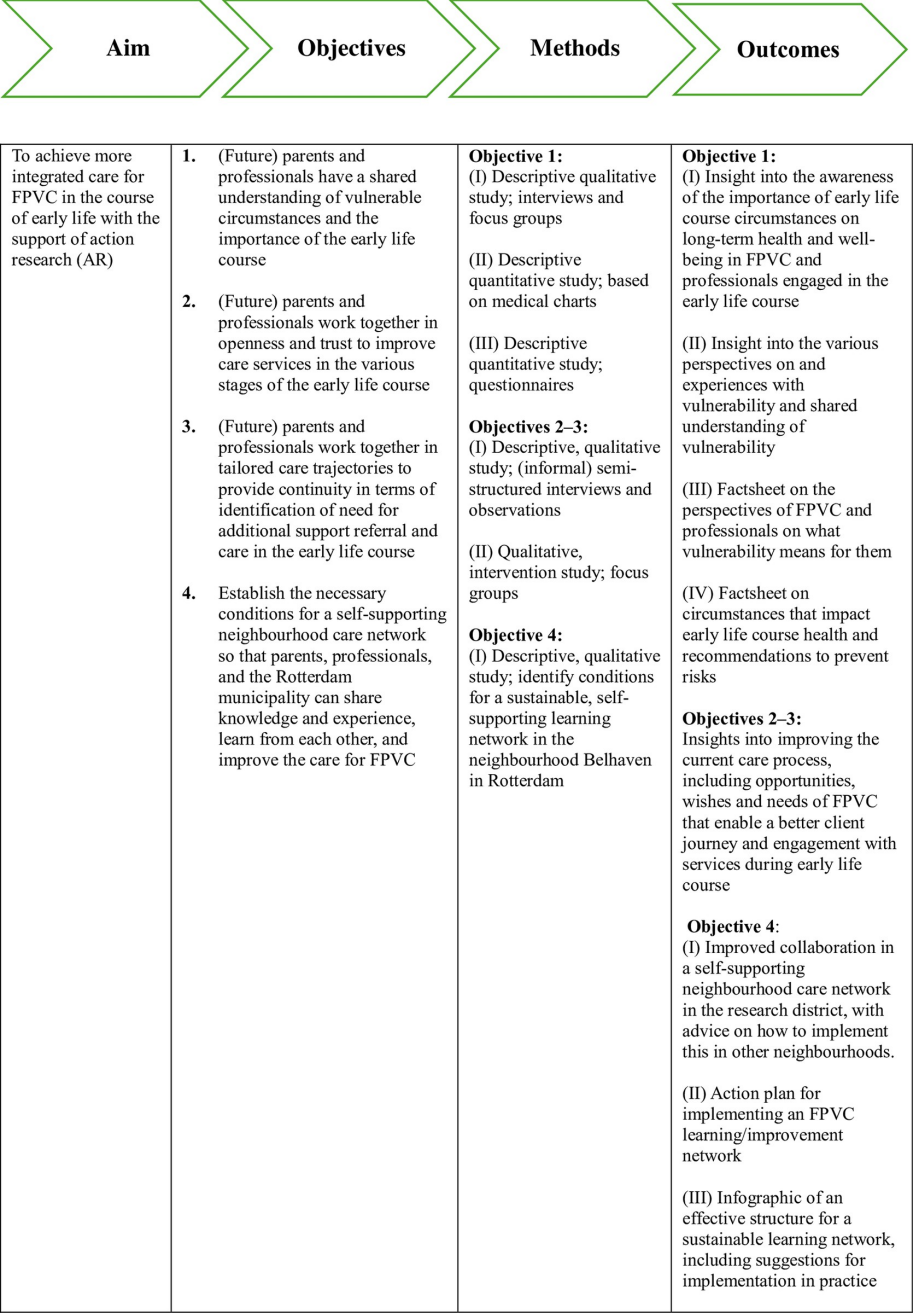
Research design.

The project will take 48 months in total. As Fig 1 illustrates, the project is split in four distinct phases, running parallel to each other. COREQ (Consolidated criteria for reporting qualitative research) guidelines will be used to ensure comprehensive reporting of the process. Also, we will comply with the Quality Action Research Checklist (QuARC), including context, quality of relationships, quality of the action research process itself and the dual outcomes [25, 26].

### Pre-phase: Joint research question articulation and a descriptive study of the lived experiences of women in vulnerable circumstances during the early life course

Prior to awarding the grant, the funder offered the opportunity for funded preliminary research for 12 months. During this pre-phase, as is common in AR, the consortium collectively formulated the research objectives in workshops with relevant actors. Moreover, as a first start, we conducted a descriptive qualitative study to explore, learn from and familiarise ourselves with the perceptions and lived experiences of women in vulnerable circumstances during the early life course. Therefore, we arranged semi-structured, in-depth interviews combined with member checking as described below. After award, two focus groups are planned with the aim of developing a profounder understanding to foster greater shared understanding between FPVC and professionals on what vulnerable circumstances entail in early life course as part of objective 1.

#### Interviews

*Recruitment and sample size*. Professionals working at one of the actor organisations invited women facing vulnerable circumstances with (wishing to have) a child in the early life phase. Purposive sampling was used to select ten women with maximum variation in care needs and demographic characteristics. Utilizing this approach enables us to capture the perspectives of a range of women who have experienced diverse forms of vulnerability during the early life course. We limited our studies to Dutch or English-speaking (future) parents, acknowledging that this may inadvertently exclude some FPVC with limited proficiency in these languages.

*Data collection*. Data was collected using semi-structured interviews as they allow for follow-up questions to gain a better understanding of the given answers. The interview guide was based on the literature and the experience of the project team whereafter they discussed the topics until a consensus is reached. To attempt to avoid interview bias and encourage conversation, the interview guide stipulated open-ended questions.

#### Focus groups

*Recruitment and sample size*. Two focus groups (with at least four participants per group) with parents and professionals will be held to discuss the themes emerging from the interviews with both FPVC in the early life course and professionals engaged this period. Professionals from the actor organisations of the consortium will use purposive sampling to invite FPVC. Also, we will invite parents from the participating parent panel. Professionals will be approached through the contacts of the consortium.

*Data collection*. Focus groups provide the opportunity for two heterogeneous groups (parents and professionals) to engage in conversation and interact with each other. The topic list for the groups will be based on the sensitising concepts: ‘vulnerability’, ‘preconception’, ‘first 1000 days’ and ‘collaboration between (future) parents and professionals during preconception and the first 1000 days’, complemented by themes arising from the interviews in the pre-phase. In order to reduce social desirability bias and establish a safe and open atmosphere for sharing (future) parents’ views and thoughts with professionals, the focus group will commence with an introductory session for the (future) parents. In this session, they will learn about the focus group process and will be assured that this setting is not only conducive to learning but where they can express their opinions without fear of judgement.

*Data analysis*. Data will be analysed using thematic content analysis from Braun and Clarke [27]. Before analysis, participants will be offered the opportunity to receive the interview transcript for verification, i.e. member check. Two researchers will start by familiarizing themselves with the data by (re-)reading the transcripts. Then both will independently generate initial codes from the transcripts and discuss them up to consensus. Any coding differences will be reconciled and again, discussed up to consensus. Once the final coding list is developed, all transcripts will be coded with the terms on this list. Finally, the codes will be merged into clusters and further defined in themes and subthemes. ATLAS-Ti will be used to facilitate data management and coding of the interviews.

### Phase 1: A descriptive study of awareness regarding the influence of early life course on adult health

In AR cycle 1, we will conduct a descriptive, quantitative, cross-sectional cohort survey to evaluate the awareness of parents and professionals regarding the influence of the early life course circumstances on adult health. The results of this study can be used to enhance (future) parents-professional collaboration by guiding professionals in effectively communicating the significance of these circumstances during consultations with FPVC, as well as for policymakers to inform efforts to disseminate this scientific knowledge to the public, ultimately aiming to improve future children’s health and well-being.

#### Recruitment and sample size

Participants, including parents and professionals, were prospectively recruited from February 18, 2022, to October 3, 2022. Preventive youth health care (one of the actors in the research collaborative), serving all children registered as Dutch citizens, facilitated the recruitment of parents. The Netherlands has an extensive and well-organised preventive youth care system, providing regular check-ups and vaccinations to pregnant women and children from pregnancy to 18 years. Parents were eligible for inclusion if they had a child aged up to two years. It is estimated that some 7500 children are born in Rotterdam every year, so the population invited to participate is approximately 15,000 parents/caregivers. Parents were excluded if they do not have sufficient command of the Dutch or English language. Professionals (N = ~300) were recruited via representatives of the actor organisations and were included if they were engaged in care during the early life course in June 2022. Written informed consent is obtained from all participants. This group of participants were selected before the data collection started.

#### Data collection

Data will be collected via a digital survey purposively designed for this study, as there is a lack of validated questionnaires. The survey will be designed in an iterative process involving both FPVC and professionals with or without a background in the field of pregnancy and early childhood to ensure relevance and usability. The survey contents will be based on both the literature to date and insights observed in the pre-phase. The survey will comprise statements related to awareness and perceptions on the importance of early life course circumstances for health and well-being during pregnancy, childhood and adulthood, applying a standard 5-point Likert scale ranging from strongly agree to strongly disagree. We will conduct a pilot phase and then the survey will be sent to parents with a child up to two years/professionals registered with the preventive youth care organisation and to professionals affiliated with the consortium.

#### Data analysis

We will use descriptive analysis models to analyse demographic data, including frequency and percentage for categorical data (e.g., education level, marital status, and years of professional experience) and means and standard deviations for continuous data (e.g., age of participants). Paired simple proportions t-tests will be used to evaluate the differences between awareness on the impact of circumstances during early life course on a child’s health during pregnancy, childhood and adulthood. Differences in awareness between parents and professionals will be evaluated with chi-squared tests. Binary logistic regression models will be used to estimate the odds ratios (OR) and 95% confidence intervals (95% CI) for the associations between characteristics of both FPVC and professionals. We will use SPSS for the statistical analyses.

### Phase 2: Inter-professional cross-domain collaboration, (future) parent-professional collaboration and continuity of care during the early life course from the perspective of FPVC and professionals

In AR cycle 2, we will conduct a descriptive qualitative study to identify 1) key elements of collaboration between FPVC and their professionals involved in early life course care, 2) key elements of (cross-domain) collaboration among their professionals, and 3) key elements of the general continuity of care. This research will teach us more about experiences with care during the early life course from the perspectives of FPVC and their involved professionals. The aim is to identify barriers and facilitators in collaboration and continuity of FPVC-care. Therefore, we will follow FPVC and their care providers longitudinally by means of observations, shadowing and (informal) semi-structured interviews.

#### Recruitment and sample size

We will employ a purposeful recruitment strategy that combines a maximum variation in vulnerability and early life phase (i.e., preconception, during pregnancy or up to two years after birth. Non-Dutch or non-English-speaking (future) parents will be excluded from the study, unless someone within their informal network can serve as an interpreter. To balance the explorative aim and include diverse cases, we estimate that 20 (future) parents will be an appropriate sample size for obtaining information richness. Professionals from organisations active in the early life course will invite parents in the pilot neighbourhood Delfshaven. The project team will distribute a video, posters and flyers to inform parents about the project. Moreover, if parental consent is obtained, a researcher will also attend the outpatient clinics with preventive youth health nurses, physicians and midwives to present the study and invite them to participate. If parents agree to participate, the researcher will do a first interview, whereafter follow-up interviews will take place. The interviews will be supplemented by participant observations of various parent-professional service encounters. If parents consent, the researcher will then ask their care providers to share their experiences around the collaboration and the patient journey in an (informal) semi-structured interview.

#### Data collection

*Observations*. Observations made on parent-professionals encounters in the field will be written up as thick descriptions, which also describe the context and thus allow for a better understanding of the observed behaviour. The number of hours that will be observed depends on the number of parents who give the researcher permission to come along to their appointments. To caution against ‘going native’, the research team will hold fortnightly reflection sessions with a senior action researcher.

*Interviews*. Data will be collected in (informal) semi-structured interviews with the option of posing follow-up questions to gain a better understanding of the answers given by FPVC and their care providers. We will develop a thematic interview guide based on the literature, discussing the topics until consensus is reached. The topics known from literature are: experience of collaboration between FPVC and care providers, experience of collaboration among care providers, therapeutic alliance and continuity of care. To avoid interview bias and encourage a conversation, open-ended questions will be used in the interview guide.

#### Data analysis

Data analysis will mirror the approach used for the interviews in the pre-phase.

### Phase 3: Fostering integrated care around identification of need for additional support, referral and care during the early life course with FPVC and professionals

In AR cycle 3, we will conduct an intervention study of FPVC and professionals working together to optimize current care trajectories for several suboptimal circumstances. In doing so, we want to learn more about the opportunities for improvement in the provision of integrated care by professionals from the medical, social and public domain. Therefore, we will hold focus group sessions.

### Recruitment and sample size

Participants will be the same professionals and FPVC as invited for AR cycle 2.

### Data collection

We will hold at least two focus group sessions as they provide the opportunity for two heterogeneous groups to engage in conversation and interact with each other. The topic list for the focus groups will be based on the literature, complemented by themes arising from the AR cycle 2 interviews and by factors from the AR cycle 1 questionnaire. At the focus group sessions, we will display a large poster visualizing the current care trajectories to serve as a guide to identifying points for improvement.

### Data analysis

Data analysis will mirror the approach used for the interviews in the pre-phase.

### Phase 4: Necessary conditions for a self-supporting neighbourhood care network

In AR cycle 6, we will conduct a descriptive qualitative study in the neighbourhood Delfshaven in Rotterdam to identify conditions for a sustainable, self-supporting learning network in which FPVC, professionals, policymakers share knowledge and experiences regarding inter-professional, cross-domain collaboration, (future) parents-professional collaboration, learn from each other and jointly improve care and guidance for FPVC. Here we will apply the ‘5C wheel’ framework of the Health Foundation [28].

#### Recruitment and sample size

Participants will be the same professionals and FPVC recruited for AR cycle 3 as well as parents from the advisory parent panel. In addition, executive professionals in the pilot district and engaged in the early life course will be invited to participate in the learning network.

#### Data collection

The ‘5C wheel’ framework describes five core features of an effective network: (1) common purpose; (2) cooperative structure; (3) critical mass; (4) collective intelligence and (5) community building. We will apply this framework to build the learning network in the pilot district and collect data in fieldnotes at community network meetings with professionals and FPVC to assess whether all five core features are visible in the network.

#### Data analysis

Data analysis will mirror the approach used for the interviews in the pre-phase.

#### Ethics approval

The Medical Ethics Committee of Erasmus Medical Centre has approved the study protocol (protocol no. MEC-2021-0692 and protocol no. MEC-2022-0296 on October 20, 2021 (part of the funding application) and May 19, 2022 respectively). Regarding the questionnaires, parents and professionals will be informed about the study aims and procedures and will be assured that participation is completely voluntary and confidential. Regarding interviews, focus groups and observations, all transcripts and field notes will be pseudo-anonymised (identifying information and names of participants will be removed and replaced by a number) to protect confidentiality. Prior to being approached for written consent, all participants (patients and professionals) will receive the project information sheet, written in Dutch language level B1 (reflecting an easy to follow use of language, according to Common European Framework of References for foreign language) in order to provide accessible information. All participants may withdraw their consent at any point of the project without having to state the reason. All actor organisations have agreed to their participation through a consortium agreement signed by the directors of the organisations. Individual care providers participate voluntarily after an informed consent procedure similar to parents.

## Discussion

### Strengths and limitations

The present study protocol is strengthened by that fact that it will generate new knowledge on how (future) parents facing suboptimal circumstances experience being called vulnerable and on which patterns of adversity are present. Moreover, the action research cycles will reveal how (future) parents facing suboptimal circumstances experience continuity of care and collaboration by *and* with professionals. The parents’ and professionals’ experiences will form the basis of improving care trajectories in collaborative learning networks. At last, relevant actors from various domains and different executive levels will take part in a learning network to share information, experiences and collaboratively improve the care for (future) parents and their child during early life course.

A limitation of the present study protocol is that like many action research studies, this research may face challenges in producing findings that can be easily generalized, given that the complexity of the issues faced by (future) parents during early life course is influenced by the specific context. As a result, the findings need to be adapted to the local situation for implementation in a different context. Also, it might be necessary to modify (parts of the) study design or add additional cycles when new information emerges from the different cycles, which necessitation such adjustments.

### Dissemination

The knowledge gained from the AR cycles will be disseminated in several ways and made available to (future) parents, (future) professionals, municipalities and policymakers on the regional and national levels to support actual change (Table 1). We will report the findings for AR cycles pre-phase-AR cycle 3 in open-access journals so that the gained knowledge is available for a wide audience. As laymen often find scientific papers difficult to read, we will write a laymen’s summary of the papers (in simple Dutch, at the B1 language level). We will post these summaries on the website for our target group and present them to the learning network.

We aim to present our findings at national and international conferences, regional and national knowledge platforms, as well as give in-person or virtual presentations at relevant educational institutions and professional associations. We will present the findings of two AR cycles on two factsheets with the ultimate aim of creating awareness. The factsheet for the pre-phase will contain information on the views of FPVC and professionals on what vulnerability in the early life course means for them. The factsheet for AR cycle 1 will contain early life course risk factors evaluated in the questionnaire and the corresponding awareness of both parents and professionals. This factsheet will also include pragmatic recommendations derived from established guidelines, detailing risk-mitigating strategies which professionals can use to educate (future) parents. We will make an infographic for AR cycle 4 that describes an effective structure of a sustainable learning network, including suggestions for implementation in practice.

## Supporting information

S1 Checklist COREQ (COnsolidated criteria for REporting Qualitative research) checklist. (PDF)

S2 Checklist (PDF)
