# Supplementary material for: Improving integrated care for (future) parents facing vulnerable circumstances in the early life course of their (future) child: An action research protocol
Source: PLoS One. 2024 Oct 31;19(10):e0305557. doi: 10.1371/journal.pone.0305557 (PMC11527274; doi:10.1371/journal.pone.0305557)
Supplement: S2 Checklist — (PDF) [file pone.0305557.s002.pdf]

Table 2 Checklist for Assessing Quality in Action Research

From: Towards a Checklist for Improving Action Research Quality in Healthcare Contexts

Factors	Questions
Context	1. Is the action driven by a practical concern?<br><br>2. Is there local, national, and international imperative influencing the practical concern?<br><br>3. Does previous research inform the practical concern?<br><br>4. Does the AR project draw on previous research?
Quality of relationships	1. Are those who have articulated the practical concern in the first instance and who also have a stake its resolution included in all the steps of the action research cycle of constructing, planning action, taking action and evaluating?<br><br>2. Can the participants be classified as co-researchers?<br><br>3. Is the level of participation of the patients/clients and coresearchers evident at each step of the action research cycle?<br><br>4. Is the AR project evaluated in terms of the quality of the relationships?
Quality of the action research process itself	1. Does the final account demonstrate a rigorous and collaborative engagement in the action research project's design?<br><br>2. Does the account demonstrate subsequent enactment of cycles of constructing, planning action, taking action and evaluating?<br><br>3. Is enactment of the cycle towards the practical and theoretical objectives transparent and reliable?<br><br>4. Is the engagement of the coresearchers evident in shared data analysis and shared reflection?
Outcomes	1. Are both forms of outcomes presented (theory (research) and action (practical)?<br><br>2. Are the outcomes sustainable?<br><br>3. Are the outcomes useful for other action research projects?<br><br>4. Does the theoretical outcome contribute to future theory development?<br><br>5. Is there a reflection on the process of engagement on the cycles by the co-researchers?
